# Combined inhibition of PARP and EZH2 for cancer treatment: Current status, opportunities, and challenges

**DOI:** 10.3389/fphar.2022.965244

**Published:** 2022-10-03

**Authors:** Xi Zhang, Xiao Huo, Hongyan Guo, Lixiang Xue

**Affiliations:** ^1^ Department of Obstetrics and Gynecology, Peking University Third Hospital, Haidian, China; ^2^ Center of Basic Medical Research, Institute of Medical Innovation and Research, Peking University Third Hospital, Haidian, China; ^3^Biobank, Peking University Third Hospital, Haidian, China

**Keywords:** PARP, EZH2, DNA damage repair, tumor immune microenvironment, tumor metabolism, PROTAC

## Abstract

Tumors with BRCA1/2 mutations or homologous recombination repair defects are sensitive to PARP inhibitors through the mechanism of synthetic lethality. Several PARP inhibitors are currently approved for ovarian, breast and pancreatic cancer in clinical practice. However, more than 40% of patients with BRCA1/2 mutations are insensitive to PARP inhibitors, which has aroused attention to the mechanism of PARP resistance and sensitization schemes. PARP inhibitor resistance is related to homologous recombination repair, stability of DNA replication forks, PARylation and epigenetic modification. Studies on epigenetics have become the hotspots of research on PARP inhibitor resistance. As an important epigenetic regulator of transcription mediated by histone methylation, EZH2 interacts with PARP through DNA homologous recombination, DNA replication, posttranslational modification, tumor immunity and other aspects. EZH2 inhibitors have been just shifting from the bench to the bedside, but the combination scheme in cancer therapy has not been fully explored yet. Recently, a revolutionary drug design combining PARP inhibitors and EZH2 inhibitors based on PROTAC techniques has shed light on the resolution of PARP inhibitor resistance. This review summarizes the interactions between EZH2 and PARP, suggests the potential PARP inhibitor sensitization effect of EZH2 inhibitors, and further discusses the potential populations that benefit from the combination of EZH2 inhibitors and PARP inhibitors.

## 1 Introduction

DNA damage repair (DDR) is a guard to maintain genome stability. DDR pathways are initiated when DNA damage occurs in cells by homologous recombination repair (HR), nonhomologous end joining (NHEJ) and DNA single-strand break repair (SSBR) (Davis and Chen, 2013; Wright et al., 2018). Poly ADP-ribose polymerase (PARP) plays a key role in DNA single-strand repair; hence, the use of PARP inhibitors in tumors with DNA double-strand repair defects blocks both DNA double-strand and single-strand repair, resulting in synthetic lethal effects and antitumor effects (Sonnenblick et al., 2015).

Clinical trials of PARP inhibitors have made continuous progress in solid tumors such as breast cancer, ovarian cancer and pancreatic cancer, but the problem of drug resistance of PARP inhibitors has gradually emerged, and one of the solutions is drug combination (Lee and Matulonis, 2020; Li et al., 2020). Preclinical studies and clinical trials focused on the combination of target drugs with PARP inhibitors include cell cycle-regulating drugs, such as the inhibitors of ATR, ATM, SHK1, SHK2, and WEE1 (Li et al., 2020), antiangiogenic drugs, such as anti-VEGF. (Bizzaro et al., 2021); immune checkpoint inhibitors, such as anti-PD-1 and anti-PD-L1, some of which have entered clinical trials but have not yet achieved advanced clinical decision. How to design a drug combination program and how to determine the best indication of the combination scheme are the propositions worth considering.

Epigenetic dysregulation has long been considered a key factor affecting tumor cell fate. EZH2 (Enhancer of Zeste homolog 2) is one of the most important epigenetic factors involved in the regulation of tumorigenesis, development and metastasis. The canonical pathway of EZH2 (Enhancer of Zeste homolog 2) catalyzes the trimethylation of histone H3K27 to silence the transcription of target genes. The noncanonical pathway includes nonhistone methylation and even transcriptional activation as well as interaction with other transcription factors (Wang and Wang, 2020). Various tumors express high levels of EZH2, which is related to advanced stage and poor prognosis (Chase and Cross, 2011; Kim and Roberts, 2016). Several EZH2 inhibitors have entered clinical trials, such as tazemetostat, GSK126, CPI-1205, PF-06821497, and SHR2554. Tazemetostat is the first EZH2 inhibitor approved by the FDA for the treatment of locally advanced or metastatic epithelial sarcoma, and relapsed or refractory follicular lymphoma (Gounder et al., 2020; Morschhauser et al., 2020). However, EZH2 inhibitors still have limited efficacy in some tumors with high expression of EZH2, such as ovarian cancer, which calls for deeper exploration of new drug combination schemes (Li et al., 2021).

Although EZH2 and PARP have distinct mechanisms and functions, respectively, both EZH2 and PARP share some common features in regulating cell fate through the cell cycle, DNA damage response, programmed cell death and other biological processes (Scott et al., 2015; Alemasova and Lavrik, 2019; Laugesen et al., 2019), which may have complex interactions. Various lines of evidence indicate that PARP and EZH2 have close crosstalk; hence, PARP inhibitors and EZH2 inhibitors may have synergistic antitumor or antagonistic effects. This review summarizes the progress on the interaction of PARP and EZH2, focusing on the aspects of DNA damage repair and the direct modification that PARP adds to EZH2, and analyzes the possible relationship between PARP and EZH2 in the tumor immune and metabolic microenvironment. In addition, advanced techniques for drug design that boost the combination of PARPi and EZH2i are also discussed.

## 2 Seesaw effect: The promotion of DNA repair by EZH2 is released by Poly ADP-ribose polymerase inhibitors

### 2.1 EZH2 is involved in the DNA damage response of tumor cells

EZH2 is one of the key factors in the response of tumor cells to DNA damage and determines their subsequent cell fate. Cells recognize DNA damage sites through two cell cycle checkpoints, namely, G1/S and G2/M, and induce cell cycle arrest, allowing cells to stay in G1 or G2 phase for DNA damage repair (Wu et al., 2011). In tumor cells with DNA double-strand damage induced by adriamycin (ADR) and etoposide (ETO), knockdown of EZH2 can mediate the deactivation of both G1/S and G2/M cell cycle checkpoints and induce apoptosis by a mechanism that depends on the presence of p53 mutations in tumor cells. Tumor cells with wild-type p53 respond to DNA damage and promote DNA damage repair through the p53-p21 pathway. FBXO32 is a target of EZH2 (Ciarapica et al., 2014; Wang et al., 2018) and is involved in mediating the degradation of the proteasome pathway of p53 downstream molecule p21; hence, the knockdown of EZH2 upregulates FBXO32 and further blocks DNA damage repair. In p53 mutant tumor cells, phosphorylation of the cell cycle checkpoint kinase ChK1 is involved in mediating the G2/M cell cycle block in response to DNA damage. EZH2 inhibitors downregulate the level of ChK1 phosphorylation through an unknown mechanism, thereby inhibiting the DNA damage response (Wu et al., 2011), and ChK1 inhibitors are more sensitive in EZH2-deficient tumor cells, more apparently inducing cell apoptosis (Leon et al., 2020).

### 2.2 EZH2 impacts DNA damage repair in tumor cells

EZH2 inhibitors harm homologous recombination repair in ovarian cancer cell lines by downregulating the expression of nonhomologous recombination repair-associated genes and thus inhibiting homologous recombination repair by treating them with EZH2 inhibitors. This mechanism of EZH2 involvement in regulating the DNA damage repair modality is CARM1-dependent (Karakashev et al., 2020). CARM1 is an arginine methyltransferase that transcriptionally represses the subunit BAF155 of the SWI/SNF complex (SWI/SNF complex), which is involved in the regulation of chromosome remodeling and is antagonistic to the PRC2 complex with EZH2 as the catalytic subunit (Kadoch et al., 2016); thus, high expression of CARM1 upregulates EZH2. In ovarian cancer cell lines with high CARM1 expression, EZH2 levels are upregulated, allowing activation of homologous recombination repair by exerting transcriptional repression on nonhomologous recombination repair-related genes, such as MAD2L2 (mitotic arrest deficient 2 like 2, MAD2L2) (Karakashev et al., 2020).

SLFN11 (schlafen family member 11) is recruited to DNA damage sites and inhibits homologous repair (Mu et al., 2016), which could sensitize the effect of DNA-damaging agents, such as PARP inhibitors and cisplatin (Stewart et al., 2017). The inactivation of SLFN11 is related to resistance to PARP inhibitors (Coussy et al., 2020), and reactivating SLFN11 by epigenetic agents could alleviate the resistance of PARP inhibitors (Murai et al., 2016; Tang et al., 2018). SLFN11 is a target gene of EZH2, and DNA damage-induced EZH2 activation suppresses the expression of SLFN11(Gardner et al., 2017). In small cell lung cancer, EZH2 inhibitors could release the expression of SLFN11, which may sensitize PARP inhibitors (Sabari et al., 2017).

In summary, EZH2 inhibitors assist PARP inhibitors in mimicking “drug-induced synthetic lethality".

### 2.3 Orchestration of EZH2 and Poly ADP-ribose polymerase in DNA damage repair in tumor cells

**FIGURE 1:**
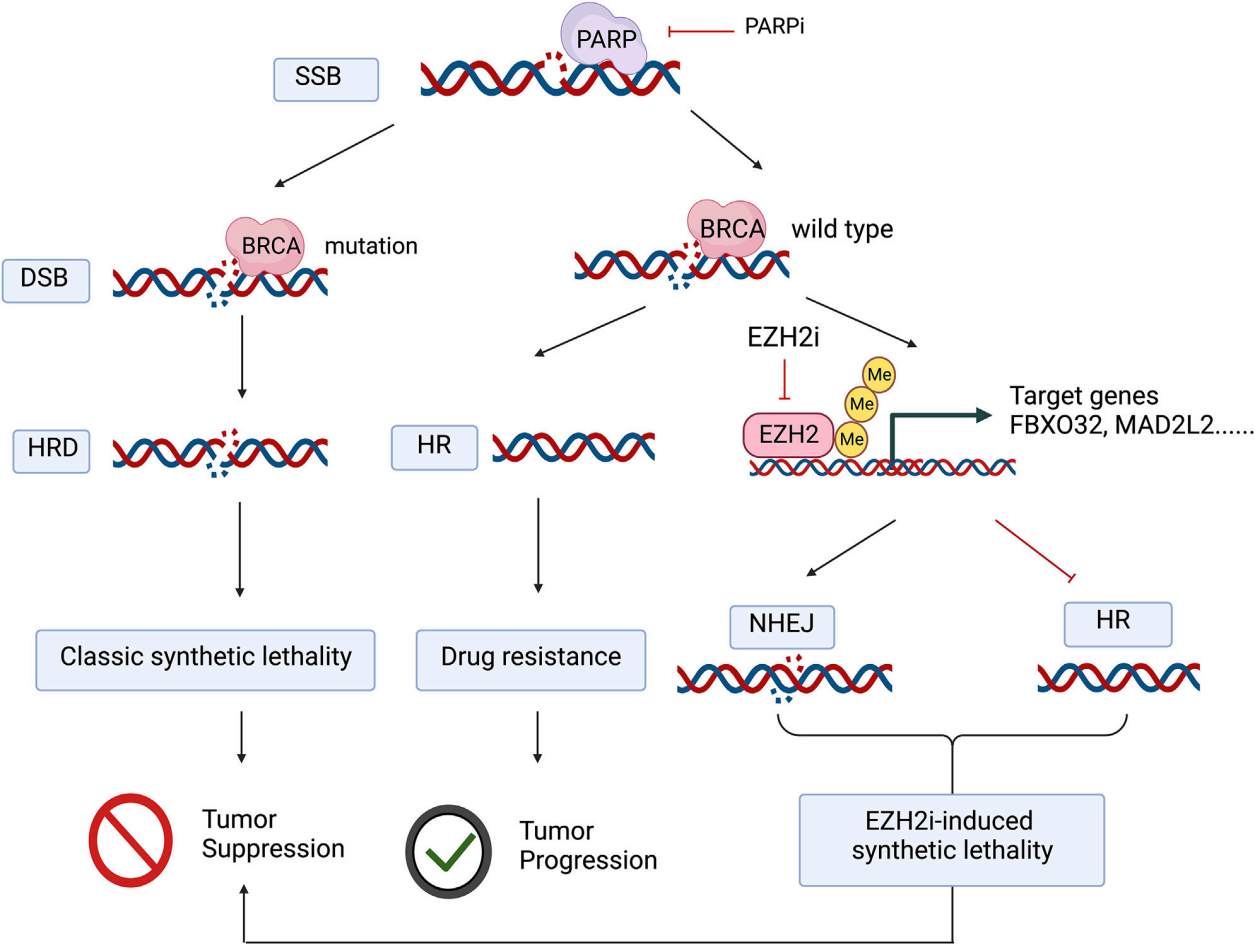
The application of PARP inhibitors in tumors with BRCA mutations blocks both DNA single-strand and double-strand damage repair, the classic synthetic lethal mechanism, whereas in BRCA wild-type tumors, even though PARP inhibitors prevent the repair of DNA single-strand breaks and further DNA double-strand breaks occur, tumor cells can still repair DNA damage due to homologous recombination repair. EZH2 inhibitors promote nonhomologous recombination repair and inhibit homologous recombination repair by deregulating histone trimethylation-mediated transcriptional repression of target genes, thus mimicking “drug-induced synthetic lethality” in concert with PARP inhibitors. Figure 1 was created with BioRender.com with a publication license.

EZH2 affects PARP-associated DNA damage repair through the canonical pathway in an H3K27me3-dependent manner. Traditionally, EZH2 acts as a histone methyltransferase catalyzing H3K27me3, which transcriptionally inactivates target genes. During the DNA damage response, EZH2 localizes to DNA damage sites in the nucleus, and this process is accompanied by the upregulation of global H3K27me3 levels (Yamamoto et al., 2019). As previously described, EZH2 affects the DNA damage repair response in tumor cells, and experiments in ovarian cancer cell lines, mouse models, and PDX models of ovarian cancer patients have verified that EZH2 promotes homologous recombination repair through H3K27me3 modification, while EZH2 inhibitor treatment prevents homologous recombination repair, thereby enhancing the antitumor effects of PARP inhibitors (Karakashev et al., 2020), which is described as a pharmacological synthetic lethal condition (e.g., Figure 1). Additionally, EZH2 also affects PARP expression levels by regulating PARP degradation. Fas-associated death domain (FADD) is a member of the tumor necrosis factor receptor superfamily that activates the downstream caspase cascade response to mediate programmed cell death (Gurung et al., 2014). EZH2 downregulates the transcriptional level of FADD, decreasing the degradation of PARP mediated by FADD and thus upregulating PARP expression (Han et al., 2020). Therefore, EZH2 inhibitors probably have the capacity to synchronize both the single-strand DNA damage-repair function and the expression of PARP simultaneously.

EZH2 may also be involved in PARP-associated DNA damage responses in tumor cells *via* a nonhistone methyltransferase catalytic pathway. In a human osteosarcoma cell line with DNA damage induced by ionizing radiation, EZH2 was recruited to the DNA double-strand damage site marked by γ-H2AX, and Suz12 and EED were also recruited to DNA damage sites as other components of the PRC2 complex; however, with PARP inhibitor treatment, EZH2 was unable to localize at the DNA double-strand damage site, suggesting that this process is PARPdependent. However, immunofluorescence revealed that these DNA damage sites did not overlap with elevated H3K27me3 markers, suggesting that EZH2 did not catalyze H3K27me3 during this process (Campbell et al., 2013). Another study showed only transient elevation of H3K27me3 at the DNA damage site, followed by rapid disappearance (Chou et al., 2010), suggesting that EZH2 may sequentially act through a noncanonical pathway in a canonical manner, but the mechanism remains to be elucidated.

Additionally, the expression of EZH2 is also affected by PARP inhibition. In a lymphoblastoid B-cell line, the inhibition of PARP reduces the expression of EZH2, followed by an elevation of global H3K27me3 (Martin et al., 2015). PARylation modified by PARP affects both the expression and activity of EZH2, which is discussed in section 3.1.

The application of PARP inhibitors in tumors with BRCA mutations blocks both DNA single-strand and double-strand damage repair, the classic synthetic lethal mechanism, whereas in BRCA wild-type tumors, even though PARP inhibitors prevent the repair of DNA single-strand breaks and further DNA double-strand breaks occur, tumor cells can still repair DNA damage due to homologous recombination repair. EZH2 inhibitors promote nonhomologous recombination repair and inhibit homologous recombination repair by deregulating histone trimethylation-mediated transcriptional repression of target genes, thus mimicking “drug-induced synthetic lethality” in concert with PARP inhibitors.

## 3 Association between EZH2 and Poly ADP-ribose polymerase in the tumor microenvironment

### 3.1 PARylation modification of EZH2 directly by Poly ADP-ribose polymerase

PARP consists of catalytic and regulatory subunits and acts as a ribosylase in posttranslational modification, using NAD^+^ as a substrate for ADP-ribose. Among the members of the PARP family (PARP1-16), PARP3, PARP6-12 and PARP14-16 catalyze mono-ADP-ribosylation of proteins, while PARP1, PARP2, PARP4, and PARP5a/b catalyze poly-ADP-ribosylation (PARylation) (Min and Im, 2020; Sanderson and Cohen, 2020). The subcellular localization determines whether PARP is involved in intranuclear events, such as epigenetic modification of DNA and histones (Ciccarone et al., 2017), DNA damage repair (Min and Im, 2020), and RNA metabolism (Ke et al., 2019), resulting in divergent cell fates, such as survival or apoptosis (Virag et al., 2013).

PARP inhibitors, including olaparib, rucaparib and niraparib, target PARP1 and PARP2, which are localized in the nucleus and are able to directly modify the PARylation of EZH2 under specific conditions. With DNA damage induced by alkylating agents in tumor cells, PARP1 reduces the affinity of EZH2 for H3K27 sites and inhibits its enzymatic activity through PARylation, while poly ADP-ribose glycohydrolase (PARG) can reverse this effect (Caruso et al., 2018). This conclusion has been confirmed by studies based on breast and ovarian cancer cell lines, where PARP1 increased the PARylation modification of EZH2 during alkylating agent-induced DNA damage or hydrogen peroxide-induced oxidative stress, further inducing the breakdown of the PRC2 complex and degradation of EZH2 (Yamaguchi et al., 2018). Thus, direct modification of EZH2 by PARP1 may be involved in regulating the response of tumor cells to DNA damage and oxidative stress, but it remains unclear which downstream signaling pathways may be altered as a result of such modifications.

### 3.2 Association between EZH2 and Poly ADP-ribose polymerase in the tumor immune microenvironment

Although studies have shown the promising combination of EZH2 inhibitors and PARP inhibitors in tumor therapy, the combination scheme of EZH2 inhibitors and PARP inhibitors does not always show antitumor effects, which may be related to the tumor immune microenvironment. Immune cells in the tumor microenvironment include CD4^+^ T cells, CD8^+^ T cells, and dendritic cells, which mainly play antitumor roles, and regulatory T cells (Tregs), as well as MDSCs with immunosuppressive effects. Additionally, tumor-associated macrophages (TAMs) play a bidirectional role in tumor immunity through the M1 and M2 polarization directions (Pantelidou et al., 2019; Ding et al., 2020; Ghonim et al., 2021; Wu et al., 2021). Immune cell infiltration in the tumor microenvironment as well as the interaction between immune cells, tumor cells and stromal cells may influence the effect of antitumor drugs, and cytokines and chemokines are involved in mediating such effects (Wu and Dai, 2017). For example, experiments in tumor-bearing mice revealed that IL-17 secreted by helper T cells (T helper 17, Th17) in the tumor microenvironment promotes upregulation of granulocyte colony stimulating factor (G-CSF) levels through the NF-κB and ERK signaling pathways, and myeloid-derived suppressive cell (Myeloid-derived suppressor cells, MDSC) infiltration increases and induces tumor resistance to anti-VEGF-targeted drugs (Chung et al., 2013).

EZH2 may affect the antitumor effects of PARP inhibitors by remodeling the tumor microenvironment. Evidence has shown that the PARP inhibition effect is dependent on the infiltration degree of T cells (Pantelidou et al., 2019; Sen et al., 2019); however, EZH2 downregulates the degree of T-cell infiltration in the tumor microenvironment (Peng et al., 2015), which hurdles the effect of PARP inhibitors. EZH2 inhibitors increase the infiltration of T cells in the tumor microenvironment (Zingg et al., 2017; Goswami et al., 2018). *In vitro* cellular assays have demonstrated that the PARP inhibitor olaparib activates the cGAS/STING pathway in triple-negative breast cancer cells, and *in vivo* experiments have further revealed that olaparib also induces the activation of the STING/TBK1/IRF3 pathway, which assists DCs in recognizing tumor antigens and further recruits and activates CD8^+^ T cells. Consistent with this, the sensitivity of olaparib was decreased in tumor-bearing mice with knockdown of CD8^+^ T cells (Pantelidou et al., 2019). Not coincidentally, EZH2 inhibitors were also able to promote STING pathway-mediated T-cell infiltration and antitumor effects (Morel et al., 2021; Xu et al., 2021), suggesting that these two inhibitors may play synergistic roles in modulating the tumor immune microenvironment.

However, the combination of EZH2 inhibitors with PARP inhibitors can also negatively affect immune cells in the tumor microenvironment, such as macrophages. Macrophages in the tumor microenvironment are divided into M1-type macrophages, which exert antitumor effects, and M2-type macrophages, which exert protumor effects, and different members of the colony-stimulating factor family are able to modulate the polarization of macrophages toward M1 or M2, respectively (Wang et al., 2014). In human breast cancer cell line MB-231 tumor-bearing mice, the combination of PARP inhibitor and EZH2 inhibitor or the simultaneous knockdown of PARP and EZH2 promoted the polarization of tumor-associated macrophages (TAMs) toward M2 and the generation of neovascularization in tumors (Yang et al., 2020).

In summary, EZH2 inhibitors upregulate the infiltration of immune cells in the tumor microenvironment and induce reprogramming of immunosuppressive cells, which enhances the antitumor efficacy of PARP inhibitors. On the other hand, the combination of these two drugs also disturbs the tumor immune microenvironment; therefore, the combination of the two drugs needs to be carefully discussed in various tumors.

### 3.3 Potential coordination between EZH2 inhibitors and Poly ADP-ribose polymerase inhibitors in tumor metabolism

Tumor cells compete with other cells in the microenvironment for metabolic materials to create a favorable microenvironment. The altered metabolic pattern of tumor cells involves glucose metabolism (Lin et al., 2020), lipid metabolism (Snaebjornsson et al., 2020), amino acid metabolism (Bott et al., 2019), etc., and drug resistance can be also induced by altered tumor metabolism under the intervention of antitumor drugs, while reprogramming of metabolism may exist as a potential new drug target (Li and Zhang, 2016).

The hub between PARP and EZH2 in metabolism may lie in NAD+/NADH and NADP+/NADPH. PARP posttranslationally modifies proteins in an NAD^+^-dependent manner, converting NAD^+^ to nicotinamide (NAM), which is synthesized by the remedial synthesis pathway to restore NAD^+^ levels. NAD^+^ is generated as NADP^+^ by the action of kinase, and the reduction products are NADH and NADPH, respectively. NAD^+^/NADH and NADP^+^/NADPH are involved in glycolysis, nucleotide synthesis and fatty acid synthesis as important cofactors (Bian et al., 2019; Navas and Carnero, 2021). EZH2 downregulates aldehyde oxidase 1 (AOX1) expression levels in a H3K27me3-dependent manner, which activates the tryptophan-kynurenine pathway and further increases the synthesis of NADP (Vantaku et al., 2020), which is the same downstream product of PARP catalysis.

Another intertwined point between PARP and EZH2 could be concluded in the aspect of lipid metabolism. Firstly, PARP and lipid metabolism are closely linked, and lipid metabolism affects PARP expression (Zhang et al., 2014; Qin et al., 2018) and enzymatic activity (Lin et al., 2008). PARP is associated with fatty acid synthesis (Szántó et al., 2021), lipid peroxidation (Bai et al., 2011; Huang et al., 2017), adipocyte differentiation (Szántó and Bai, 2020), and other lipid metabolic processes. Note worthily, these processes can affect the expression and activity of EZH2 or are regulated by EZH2 (Zhang et al., 2022). RNAseq and proteomic data suggest that the PARP inhibitor olaparib causes cells to undergo altered lipid metabolism, highlighted by processes such as fatty acid biosynthesis and fatty acid *ß*-oxidation (Mehta et al., 2021). The expression of sterol regulatory element-binding protein 1 (SREBP1), a transcription factor that promotes lipid synthesis, especially cholesterol synthesis is downregulated by posttranslational modification of PARP, and knockdown of PARP or treatment with PARP inhibitors both promote hepatic lipid accumulation (Szanto et al., 2014; Pang et al., 2018).

Secondly, PARP is able to alter the ratio of polyunsaturated fatty acid (PUFA) composition in skin tissues. For example, DHA (docosahexaenoic acid) and EPA (eicosapentaenoic acid) levels can be upregulated and further leads to the formation of a proinflammatory microenvironment (Kiss et al., 2015). At this point, it was also found that applying a certain type of EZH2 inhibitor or shRNA-EZH2 knockdown approach, PUFA was upregulated in multiple solid tumor cell lines and animal models (Zhang et al., 2022). Moreover, in breast cancer cells, ω-3 polyunsaturated fatty acids such as DHA and EPA induce the degradation of EZH2 by the proteasome pathway, thereby downregulating EZH2 protein levels and alleviating the transcriptional repression of EZH2 target genes such as E-cadherin and IGFBP3(Dimri et al., 2010).

Thirdly, as a key regulator family in lipid metabolism, peroxisome proliferator-activated receptor (PPAR) is a member of the intranuclear receptor transcription factor superfamily, which mainly includes PPARα, PPARβ and PPARγ(Wang et al., 2020). PPARα is mainly expressed in hepatocytes, cardiomyocytes and brown adipocytes. PPARα target genes are key enzymes for fatty acid oxidation, so PPARα has an important role in regulating fatty acid oxidation (Kersten, 2014). The EZH2-PPARγ axis has been confirmed to promote cancer proliferation (Hu et al., 2021). The posttranslational modification of PPARα by PARP and SIRT1 (Sirtuin) has a competitive effect, and PARP prevents the binding of PPARα to the promoter region of fatty acid oxidation-related genes through PARylation modification, while SIRT1 catalyzes deacetylation to promote the localization of PPARα in the promoter region of target genes, thereby inhibiting fatty acid oxidation (Huang et al., 2017). It has been suggested that the deacetylation of EZH2 by SIRT1 inhibits the binding of EZH2 to its target genes and weakens the pro-carcinogenic effect of EZH2 (Wan et al., 2015); on the other hand, knockdown or inhibition of EZH2 can promote the expression of SIRT1. It can be speculated that there may be an intersection between EZH2 and PARP-induced downstream metabolism-related changes and vice versa.

At last point, both PARP and EZH2 are involved in the adipocytes differentiation and the formation of lipid droplet, respectively. Adipocytes eventually differentiate into brown, white and beige adipocytes. Adipocyte terminal differentiation-related genes are regulated by PPARγ. PPARγ is mainly expressed in adipose tissue (Cristancho and Lazar, 2011) and promotes adipocyte differentiation by forming a positive feedback loop with C/EBPα (Cebpb CCAAT/enhancer binding protein) (Farmer, 2006). During adipocyte differentiation, PARP was recruited to the promoter regions of PPARγ2 target genes such as CD36 and aP2 to promote the expression of both genes. This process was accompanied by the downregulation of H3K9me3 and upregulation of H3K4me3 in the promoter region of PPARγ2 (Erener et al., 2012), suggesting that PARP may be involved in regulating adipocyte differentiation by affecting epigenetic modifications. In line with this phenomenon, EZH2 coincides with the ability to catalyze histone methylation in the promoter region of PPARγ2, the result of which promotes processes such as liver fibrosis (Du et al., 2021) and pancreatic cancer cell proliferation (Hu et al., 2021). Interestingly, our previous research has also found EZH2 inhibitor GSK126 upregulates the expression level of fatty acid synthesis related genes and results in lipid droplet accumulation in liver (Zhang et al., 2022). Hence, although there is still a lack of direct evidence between PARP and EZH2 in regulating or being regulated in lipid metabolic processes, several hints have indicated the possibility of an interaction between them in the given circumstance.

### 3.4 The limitation of the combined strategy due to tumor-suppressor role of EZH2

Beyond the traditional oncogenic role, EZH2 also acts as a tumor-suppressor in certain condition, which possibly brings limitation to the combined strategy (Gan et al., 2018). In Kras-driven lung adenocarcinoma mouse model, loss of *Ezh2* release the insulin-like growth factor 1 (Igf1), further amplify the activation of Akt-ERK signaling and promote tumor formation (Wang et al., 2017). In pediatric high-grade gliomas, specifically diffuse midline gliomas (DMG) which is characterized by the signature K27M mutation in histone H3, EZH2 ablation promotes tumor cell proliferation, while EZH2 overexpression reverses this effect in H3WT DMG mouse models (Dhar et al., 2022). Therefore, the combination strategy should be cautiously evaluated in different type of tumors due to the double-edge effect of EZH2.

## 4 Preclinical studies of combination strategy of EZH2 inhibitors and Poly ADP-ribose polymerase inhibitors

The regimens of EZH2 inhibitors in combination with other drugs include those with chemotherapeutic agents, immunotherapy and targeted therapies. The combined effects of EZH2 inhibitors and PARP inhibitors are summarized in Table 1 and vary in different cancer types. Currently, experiments and clinical trials raise a question worthy of consideration: which population benefits more from a combination of EZH2 inhibitors and PARP inhibitors? The BRCA mutation state is a distinguishing feature, and the effect of combination rules still relies on the state of BRCA deficiency (Wicha, 2009; Schlacher, 2017; Chen, 2021); however, studies in ovarian cancer suggest that EZH2 inhibitors sensitize PARP inhibitors in CARM1-high patients and that the CARM1-high population highly overlaps with wild-type BRCA, suggesting that EZH2 inhibitors are expected to be an effective drug combination regimen for PARP inhibitors in specific ovarian cancer patient groups, further expanding the applicability of PARP (Hatchi and Livingston, 2020; Karakashev and Zhang, 2020).

**TABLE 1:** EZH2 inhibitors in combination with PARP inhibitors in pre-clinical researches.

PARPi + EZH2i	EZH2 target gene	Cancer	Genetic characters	Material	Methods	Results	Year
Olaparib + EZH2 siRNA	β-catenin	Ovarian cancer	BRCA1/2^wild^	Ovarian cancer cell line HeyA8	CCK8	si-EZH2 increases the sensitivity of Olaparib by regulating *ß*-catenin signal pathway	2021Sun et al., 2021
Olaparib + GSK126	MAD2L2	Ovarian cancer	CARM1^high^BRCA1/2^wild^	CARM1^high^ A1847 and CARM1^KO^ A1847 cell line; subcutaneous xenograft mice models	colony formation assays	Olaparib and GSK126 show synergistic effect in suppressing CARM1-high *in vitro* and *in vivo*	2020 Karakashev et al., 2020
				CARM1^high^and CARM1^low^ patient-Derived Xenografts mice models	Xenograft Models	The mechanism is that EZH2 inhibition induces MAD2L2 expression and non-homologous end-joining	
Dual PARP and EZH2 inhibitor	—	Breast cancer	ER (-)PR (-)HER2(-)BRCA1/2^wild^	TNBC cell lines MDA-MB-231 and MDA-MB-468	MTT assay	Dual target agent shows better inhibitory activity than single agent of Olaparib or EZH2, and their combined treatment	2021 Wang et al., 2021
Olaparib + GSK126, PARP^KO^ + EZH2^KO^	RELA/B	Breast cancer	ER (-)PR (-)HER2(-)BRCA1/2^wild^	TNBC cell lines MDA-MB-231	CellTiter-Glo Assay Kit, colony formation assays	PARP1-PRC2 double depletion, and combined administration of Olaparib and GSK126 promotes cancer growth	2020 Yang et al., 2020
Olaparib + GSK343	HOXA9, DAB2IP	Breast cancer	EZH2^high^BRCA^mut^	BRCA^mut^ cell lines SUM149, MDA-MD-436 and UWB1.289	Colony formation assay and soft agar assay	EZH2 inhibitor sensitizes PARP inhibitor in BRCA^mut^ cell lines	2018 Yamaguchi et al., 2018
Olaparib + GSK126	MUS81	Breast cancer	BRCA2^−/−^	HeLa, VU423 (BRCA2^−/−^), A2780, U2OS, HEK 293T cell lines; KB2P PARPi-naïve tumor-bearing mice model	Clonogenic survival assay; Xenograft Models	EZH2 inhibitor promotes PARP inhibitor resistance by stop recruiting MUS81 and cause fork stabilization	2017 Rondinelli et al., 2017
Olaparib + UNC1999	—	Acute myeloid leukemia	BRCA1^−/−^	LCLs, HeLa and HEK293 cell lines	Bio Rad TC20 Automated Cell Counter	EZH2 inhibitor sensitizes PARP inhibitor in BRCA cells	2018 Caruso et al., 2018
				BRCA1-mutated and BRCA1-reconstituted MDA-MB-436 cell lines			

It is noteworthy that in certain genetically characterized populations, the combination of EZH2 inhibitors with PARP inhibitors shows negative effects. In BRCA2-deficient breast cancer cell lines, the combination of the EZH2 inhibitor GSK126 and the PARP inhibitor rucaparib diminishes the single-agent antitumor effect of the latter. A similar effect is confirmed in animal model, that EZH2 inhibitors induce PARP inhibitor resistance (Rondinelli et al., 2017). The EZH2 inhibitor interferes with the localization of MUS81 to replication forks, thereby enhancing DNA stability and further inducing PARP inhibitor resistance.

In addition, the drug structure may also affect the effect of the combination of the two drugs, and how to design the structure of the targeted drug is a proposition worthy of consideration. An ideal drug is to inhibit the enzyme activity along with the protein levels of both PARP and EZH2, and this concept is expected to be realized by proteolysis-targeting chimera (PROTAC). By anchoring the ubiquitin ligase E7 at the appropriate site of the EZH2 inhibitor GSK126 molecule by PROTAC technology, EZH2 enzyme activity was inhibited while inducing the degradation of its proteasome pathway, and the levels of other components of the PRC2 complex, such as SUZ12 and EED, were simultaneously downregulated (Liu et al., 2021). Adding a linker connected to the EZH2 inhibitor EPZ6438 to E3 ligase systems also shows a profound effect (Tu et al., 2021). Therefore, both the canonical and noncanonical oncogenic pathways of EZH2 are blocked by the PROTAC strategy (Wang et al., 2022). A novel PARP inhibitor anchored by ubiquitin ligase E3, designed based on PROTAC technology, has also been reported to have a significantly lower IC50 than the conventional PARP inhibitor Niraparib (Zhao et al., 2019). In addition, a new compound as dual-target inhibitor of PARP and EZH2, synthesized on the basis of olaparib and tazemetostat by linking the two drug molecules through hydrogen bonding, inhibited tumors 15–80 times more effectively than the PARP inhibitor alone in a BRCA wild-type triple-negative breast cancer cell line (Wang et al., 2021).

## 5 Perspectives

With the development of PARP clinical trials in ovarian cancer, studies related to drug resistance to PARP inhibitors are gradually receiving attention. Clinical studies of PARP in combination with other drugs have focused on kinase inhibitors, WEE inhibitors, immunotherapy, and other drugs, and epigenetic drugs may play an important role as potential drug combination solutions. However, the following aspects are noteworthy:

First, how to design the drug combination regimen. The combined dose and administration method of two or more drugs are prominent factors that may affect their effects. Second, how to determine the best population for the combination of drugs. Both PARP and EZH2 have a wide range of biological functions in addition to acting on tumor cells, such as being involved in various cells, including immune cells and adipocytes, and they also have a regulatory effect on tumor metabolism and immune function. EZH2 acts both tumor-promoter and tumor-suppressor roles in certain type of cancer. Therefore, it is necessary to comprehensively evaluate the molecular characteristics, immune typing, and metabolic typing of tumors to confirm the indications for combination therapy. Third, the design of new drugs based on existing drug combinations is promising. The most excited advance mentioned above has been reported lately that the compound with dual PARP and EZH2 inhibitors, showed good inhibitory activity against PARP-1 and EZH2 and good inhibitory effects on multiple type of triple-negative breast cancer (TNBC) cell lines with wild-type BRCA, with a slight harm on normal cells, suggesting possible safety of the combined strategy in clinical context (Wang et al., 2021). However, the evidences *in vivo* are still required. Besides, simultaneous inhibition of enzyme activity and protein expression will be considered in the future to improve the efficacy of tumor treatment. Fourth, targeting drug delivery and enrichment is a proposition that deserves further exploration. Both EZH2 and PARP1 are multitargets, so targeting tumor cells through novel drug loading and delivery systems is one of the solutions to improve the existing therapeutic efficacy in this case. The current nanodelivery, targeted loading, and delivery systems that target tumor cells, indicating markers, offer technical possibilities for this strategy.

## Abbreviations

SSB, Single strand break; DSB, Double strand break; HRD, Homologous repair defect; HR, Homologous repair; NHEJ, Non-homologous end joining.
