# Supplementary material for: Combined inhibition of PARP and EZH2 for cancer treatment: Current status, opportunities, and challenges
Source: Front Pharmacol. 2022 Oct 3;13:965244. doi: 10.3389/fphar.2022.965244 (PMC9574044; doi:10.3389/fphar.2022.965244)
Supplement: Supplementary file 1 [file Table1.docx]

| **PARPi＋EZH2i** | **EZH2 target gene** | **Cancer** | **Genetic characters** | **Material** | **Methods** | **Results** | **Year** |
| --- | --- | --- | --- | --- | --- | --- | --- |
| Olaparib＋EZH2 siRNA | β-catenin | Ovarian cancer | BRCA1/2^wild^ | Ovarian cancer cell line HeyA8 | CCK8 | si-EZH2 increases the sensitivity of Olaparib by regulating β-catenin signal pathway. | 2021(Sun et al., 2021) |
| Olaparib＋GSK126 | MAD2L2 | Ovarian cancer | CARM1^high^BRCA1/2^wild^ | CARM1^high^ A1847 and CARM1^KO^ A1847 cell line; subcutaneous xenograft mice models;  CARM1^high^and CARM1^low^ patient-Derived Xenografts mice  models | colony formation assays;  Xenograft Models | Olaparib and GSK126 show synergistic effect in suppressing CARM1-high *in vitro* and *in vivo*.  The mechanism is that EZH2 inhibition induces MAD2L2 expression and non-homologous end-joining. | 2020(Karakashev et al., 2020) |
| Dual PARP and EZH2 inhibitor | - | Breast cancer | ER(-)PR(-)HER2(-)BRCA1/2^wild^ | TNBC cell lines MDA-MB-231 and MDA-MB-468 | MTT assay | Dual target agent shows better inhibitory activity than single agent of Olaparib or EZH2, and their combined treatment. | 2021(Wang et al., 2021b) |
| Olaparib＋GSK126, PARP^KO^+EZH2^KO^ | RELA/B | Breast cancer | ER(-)PR(-)HER2(-)BRCA1/2^wild^ | TNBC cell lines MDA-MB-231 | CellTiter-Glo Assay Kit, colony formation assays | PARP1-PRC2 double depletion, and combined administration of Olaparib and GSK126 promotes cancer growth. | 2020(Yang et al., 2020) |
| Olaparib＋GSK343 | HOXA9, DAB2IP | Breast cancer | EZH2^high^BRCA^mut^ | BRCA^mut^ cell lines SUM149, MDA-MD-436 and UWB1.289 | Colony formation assay and soft agar assay | EZH2 inhibitor sensitizes PARP nhibitor. | 2018(Yamaguchi et al., 2018) |
| Olaparib＋GSK126 | MUS81 | Breast cancer | BRCA2^-/-^ | HeLa, VU423 (BRCA2−/−), A2780, U2OS, HEK 293T cell lines; KB2P PARPi-naïve tumor-bearing mice model | Clonogenic survival assay; Xenograft Models | EZH2 inhibitor promotes PARP inhibitor resistance by stop recruiting MUS81 and cause fork stabilization. | 2017(Rondinelli et al., 2017) |
| Olaparib＋UNC1999 | - | Acute myeloid leukemia | BRCA1^-/-^ | LCLs, HeLa and HEK293 cell lines;  BRCA1-mutated and BRCA1-reconstituted MDA-MB-436 cell lines | Bio Rad TC20 Automated Cell Counter | EZH2 inhibitor sensitizes PARP inhibitor in BRCA cells. | 2018(Caruso et al., 2018) |

Table 1 EZH2 inhibitors in combination with PARP inhibitors in pre-clinical researches
